# Supplementary material for: Patterns of Intron Gain and Loss in Fungi
Source: PLoS Biol. 2004 Nov 30;2(12):e422. doi: 10.1371/journal.pbio.0020422 (PMC532390; doi:10.1371/journal.pbio.0020422)
Supplement: Table S1 — Also available at http://genes.mit.edu/NielsenEtAl/. (4.3 MB ZIP). [file pbio.0020422.st001.zip › NielsenEtAl/html/1431.html]

AN8286.1.NCU02626.1.MG04366.1.FG01004.1


```
 CLUSTAL W (1.82) Multiple Sequence Alignments - Introns Inserted


Sequence 1: MG04366.1	972 aa
Sequence 2: FG01004.1	939 aa
Sequence 3: NCU02626.1	938 aa
Sequence 4: AN8286.1	889 aa
Alignment Length: 1054 aa
Number Identitical Residues: 399 aa
Alignment Score (without introns) 17844


MG04366.1 	--MSTTTLPRAANGPPTSALPALPVGKTRK----AAGGIVAPS---RSASSTPRSG-LRA
NCU02626.1	MSSTVPKPARLSSGPPNSAGPALSMGKTRR----SIGGLSVSTN--TAMPSTPKSGVLRT
FG01004.1 	-----------------MALPALPVNKTRK----SKGSLPTHTSSIKSVPGTPRSG-LRA
AN8286.1  	-MSTLPQPSRTASGPPLGAAAQLPIPKSRKSLPTSVESPRAVSPSKLRTPSSPRPTLSKS
          	   : .  .  :....  * . *.: *:*:: .::  .  . : :    ..:*:.   ::

MG04366.1 	P-STSFLPPSTPAPTT--ALPQPRSVSGIVGPGTKTIRKTVSINSFPQPPRGDSG---RA
NCU02626.1	PSSASNFRNSTASPATGGGLPQPRAVSSIN-ASSKTLRKSVSINSFPQPPRGEGR---LT
FG01004.1 	PSSTTNLTPSGPAPTA--ALPQP-----------KTLRKSVSINSFPQPPRGDTR---TN
AN8286.1  	PLSNSATNISAARSTTVARTPSS---------PDKSLRRTISIAAFPQPPRPGSRPSTAS
          	* * :    * . .:: .  *..           *::*:::** :******    .::  

MG04366.1 	S-LPPSPLSSGASAARRPK---TAISTDTHTSGNTPSLLNGSEGIKSVRNAGARSSDGFI
NCU02626.1	S-LPPSPLSAVAS--SRKSK--TPTTPTYQFSGSTGSLVNGSGDGRTAAR--SRNSDGLI
FG01004.1 	SGVPPSPRTSDRPRPSRKSSKPTKESMYSTFSSNTPSFLNGSGDGQSINN--VRMSDGLI
AN8286.1  	SMTAPHGLQSSGSVKSKRGSR-PSAGTTSQRSSKTHSLLSVG-----------AAVPGTD
          	*  .*    :  .   :  .  .        *..* *::. .               *  

MG04366.1 	SVGSPPQSRSSSAQDSYSTSATQYDDMTDGTQQKSGAGGESKRTSKIDGKGNVIVSVRVR
NCU02626.1	SIASPPQSRSSSAQDSYSTSATQYEDMEATQS---------------------------R
FG01004.1 	SVASPPQSRSSSAQDSYSTSATTYDDPADGPGQKSGVSAD-KRASKQDGKGNVVVSVRVR
AN8286.1  	MEASPSQSRSSSAEGSYSTSATTFDEGEPASTKS------------KETKGNVIVSVRVR
          	  .**.*******:.******* :::      ..             . ...   :   *

MG04366.1 	PDAGSNDHGRTE~GEWMVDGRRSLISYRGKEGGDYFYD1NVFATHDDNSKVYDHSAKRLV
NCU02626.1	PDTSAG---SKN1GEWMVDGRRSLISYNGKEGGDYYYG1---------------------
FG01004.1 	PDANGN-NGSPD~GEWMVDGRKSLISFRGKDGGDHYYD1NVFTTHDHNSRVYDHIAKRLV
AN8286.1  	PNVGGE--SSAN~PEWIVDARRGLLVYNGKEGGDYYYD1NVFSAMENNARVYDSAAKRLV
          	*:...   .  :  **:**.*:.*: :.**:***::*. .  :: . .:   .  :.   

MG04366.1 	RRVMEGYHGTVFAYGMTGTGKTFSMQGTASSPGVIPLAITDIFSYIRETPSREFLLRVSY
NCU02626.1	--------------------KTFSMQGTASSPGVIPLAITDIFSYIRETPSREFLLRVSY
FG01004.1 	RRVMEGYHGTVFAYGMTGTGKTFSMQGTASSPGVIPLAITDIFSYIRETPSREFLLRVSY
AN8286.1  	RRVMEGYHGTVFAYGMTGTGKTFSMQGTATSPGVIPLAITDIFSFIRETPHREFLLRVSY
          	    ..  .:  : . :.:.*********:**************:***** *********

MG04366.1 	LEIYNERIHDLLSMPTGGV-GGNTAQEEIKLREDSKRGVYASPLKEEIVQSPTQLLRVIA
NCU02626.1	LEIYNERIHDLLSMATGGNGPGAPAQEEIKLREDSKRGVYATPLKEEIVQSPTQLLRVIA
FG01004.1 	LEIYNEKIHDLLSMSTS---GGAPQQEEIKLREDSKRGVYATPLKEEIVQSPTQLLRVIA
AN8286.1  	LEIYNEKIHDLLSASTG------SSSEDIKLREDSKRGVYATPLKEEIVQSPTQLLRVIA
          	******:****** .*.      . .*:*************:******************

MG04366.1 	RGDQARRTASTQFNARSSRSHAVVQIVVESRERVPGTATPGGDSKRSGMLPGGVRVSTLS
NCU02626.1	RGDQARRVASTQFNARSSRSHAVVQIVVESRERVPAGDSSGG-SKRQGLLPGGVRVSTLS
FG01004.1 	RGDQARRTASTQFNARSSRSHAVVQIVVESRERMPG-GAAAAEGKRSGLLPGGVRVSTLS
AN8286.1  	RGDHARRTGSTQFNARSSRSHAVVQIVVESRERVPTGTTQDR---RSGLAPGGVRVSTLS
          	***:***..************************:*   :      *.*: **********

MG04366.1 	LIDLAGSEKAAESKERRTEGSHINKSLLTLGTVISKLSEYKDKDGKMSDKDGKHLPYRDS
NCU02626.1	LIDLAGSEKAADTKERRQEGSHINKSLLTLGTVIAKLSENK-------DKDDKHLPYRDS
FG01004.1 	LIDLAGSEKAAESKERRQEGAHINKSLLTLGTVISKLSEWKEKEAKGGDKEGKHLPYRDS
AN8286.1  	LIDLAGSERAADDKERRTEGAHINKSLLTLGNIISRLSETKG--KTAADKDGKHLPFRDS
          	********:**: **** **:**********.:*::*** *    . .**:.****:***

MG04366.1 	KLTRLLQGALSGNSLVSILCTIQTGAGGSVAGGNTHVSETINTLKFAARAKNSIVSHAKK
NCU02626.1	KLTRLLQGALSGNSLVSILCTIQCGAGGSTAGS-THINETLNTLKFASRAKNSIVSRAKR
FG01004.1 	KLTRLLQGALSGNSLVSILCTIQIGAGNSAVLANNHTLETINTLKFASRAKNSIISHAKK
AN8286.1  	KLTRLLQPALSGNSLVSILCTVQLSS--------LVASETLNTLKFAARAKNNIVSHAKR
          	******* *************:* .:            **:******:****.*:*:**:

MG04366.1 	AEEALG-AGGEGGARVLLERYRMEIQELKSQLNSQAKGNKGKDTEEDKTKDVEEEKAREK
NCU02626.1	ADEALG-AGGDGNARVLLERYRMEIAELRKELEKQAKANSQKEADEEMERDAKEELAREL
FG01004.1 	AEEALG-AGGEGGARVLLERYRMEITELRQQLEAQAKKKKS-DVEEEPVLDEEEEKAREA
AN8286.1  	AEEAFGGGGGDAGSRVLLERYRMEIQALRCQLENQTKAQAEKELKLE-------EQKLEQ
          	*:**:*..**:..:***********  *: :*: *:* :  .: . :       *   * 

MG04366.1 	EAEHRHEEQLLEMQLARTALKERIDHLNRLILSSKSTGVNSNGSYSSLGMHPRSS-----
NCU02626.1	EMEQRHEEQMLEMQLARTALKERIDHLNRLILSSKSIGVNANGSLSSYSLNTFNNRFS--
FG01004.1 	QAAERHEEQILEMQLARTALKERIDHLNRLILSSKSIGVNTNGTMSALGQYSRFSQFSQF
AN8286.1  	EAQERHEEQMLEMQLARTALKERIEHLNRLILSSKSTGVNSQGAMSALGRLSRLS-----
          	:  .*****:**************:*********** ***::*: *: .  .  .     

MG04366.1 	-----LVSVRSMGTTS--GRSLLERTSSMTSSSSTLGRRTSSRRVSSS--VGEMNVEED-
NCU02626.1	-----VGSVRSVMTASNAGRPFLERSTSMASSSSTIGRRSFEKRSSSTGPEAPLTEAEDE
FG01004.1 	SQISLPASIRSSVATSSGGRPLVERTASMTSASSTIGRRSSGGQKNSG--DNTPTEVED-
AN8286.1  	----------SIDPGS--------RSLRSSVSQSTLGTSMRPVSFLSVHSQEFHLSNEDE
          	          *  . *        *:   : :.**:*         *  .       **.

MG04366.1 	DSHGEYGDGVASLAAQNRALQADLTDKNRYIATLEKRLLQARRTSSARASVGMGGS-KGI
NCU02626.1	DSIGEFGDGTASLAAQNRALQADLADKNRYIQTLEKRLLQARRASSSRTSLGITPG-NKI
FG01004.1 	DSAGEFGDGTASLTAQNRALQADLVDKNRYIATLEKRLLQARRASSSRASVGFAAPNKAI
AN8286.1  	DTIGEFADGKASAQRQIAALQADLADKNRYIATLERRLLQARRSSHSRMSLGIKAN----
          	*: **:.** **   *  ******.****** ***:*******:* :* *:*:       

MG04366.1 	MVGEDHSVATVIKEKDAEIAELRARLDDKDRMLSALRSAARSRDNADRVESRTEMRTSQI
NCU02626.1	LVGEDHSVSTLLREKDAEIIELRARLEDKDRMLAALRSAARSRDNADRVESRTSNR----
FG01004.1 	MVGEDHSVSAALKEKDTEIADLRARLDDKDRMLAALRSAARSRD----------------
AN8286.1  	SSTDNPNFVAQLREKDMEINELRLQLDDKDRMLTALRSAARHRD----------------
          	   :: .. : ::*** ** :** :*:******:******* **                

MG04366.1 	MDGSAKSPSEPRTSQIMEGLAKPKSPLGSPPSEAAPSPPSLARQMSGIKKRTKSVDEMSR
NCU02626.1	---NSRVFSDDGNYSLAN---TPGLTTLSSPTGSTPTLSSQVSLMNSIKTRTKSVDEMSK
FG01004.1 	---TAEATVEPRS---------PLSPK-PAVQESSSEKPGSAKTAVTEKKRTRGVDEMNN
AN8286.1  	---LAQLTLDNQS-----------LPKETSSPDREPPPTSQAPDPGAKR---KSMDEVSR
          	    :.   :  .            .  ..     .  .. .      :   :.:**:..

MG04366.1 	ILDEM0IQDRVESGQIVRGVRGSVRIASER--KAPAIPADDQLM~---------------
NCU02626.1	MLDDM~IQDRVESGQLIRGNRGSIRVAPTPAPTMPPPPPPGPIH~GEHREPIRLSQRTPS
FG01004.1 	LLDEM~LQDRVEKGQVVRGKRGSVRLAVGQNMDSLAEPNLEPLR~---------------
AN8286.1  	ILDEM~IQDRVESGHLIKGARGSVRVAPGSRRASESHQAAGPTG1--------------A
          	:**:* :*****.*::::* ***:*:*        .                       :

MG04366.1 	HEPKP-------ALALQV--------------------
NCU02626.1	RTPSPPTQMNEPPIKLQLLSKLRTSYVAESQGPVIMEV
FG01004.1 	RTPTPNPPEDRNDVSVEA--------------------
AN8286.1  	MTNLP---------RIDA--------------------
          	    *          ::
```
